# Utilization of a HIPAA-compliant large language model chatbot in an academic pediatric medical center

**DOI:** 10.1371/journal.pdig.0001684

**Published:** 2026-09-17

**Authors:** Benjamin Rader, Dinesh K. Rai, Melissa L. Stewart, Sarah Lejfer, Sampath Chennuri, James Hartwell, Victor Moyano, Max H. Witwer, Peter C. Laussen, Heather L. Nelson, John S. Brownstein

**Affiliations:** 1 Innovation & Digital Health Accelerator, Boston Children’s Hospital, Boston, Massachusetts, United States of America; 2 Department of Anesthesiology, Critical Care and Pain Medicine, Boston Children’s Hospital, Boston, Massachusetts, United States of America; 3 Department of Anesthesia, Harvard Medical School, Boston, Massachusetts, United States of America; 4 Executive Vice President of Health Affairs, Boston Children’s Hospital, Boston, Massachusetts, United States of America; 5 Chief Information Officer, Boston Children’s Hospital, Boston, Massachusetts, United States of America; 6 Department of Pediatrics, Harvard Medical School, Boston, Massachusetts, United States of America; 7 Department of Biomedical Informatics, Harvard Medical School, Boston, Massachusetts, United States of America; Australian Institute of Health Innovation, AUSTRALIA

## Abstract

Despite growing interest in LLM chatbots, little evidence exists on usage patterns in hospital systems. This paper presents a mixed-methods case study of “InternalGPT,” a HIPAA-compliant LLM chatbot made available to employees of the institution. Survey responses and 14 months of system utilization data were analyzed across diverse professional roles in the institution. Of approximately 15,800 hospital employees, 2,149 (13.6%) requested InternalGPT access. Motivations for sign-up aligned with professional roles: operations professionals prioritized administrative automation, clinicians focused on clinical augmentation, and scientists on research and coding. Among InternalGPT users, 52.8% recorded at least one token use; 33.6% never logged-in, and 13.7% logged-in but never used the tool. The top 20% of users consumed 69.4% of tokens. Key barriers to engagement among non-users included limited time for exploration (51.4%) and difficulty using (21.8%), integrating (15.5%), or accessing (9.2%) InternalGPT. Among a small group of employees with sustained InternalGPT use (n = 461), a subset who responded to a self-report survey (n = 92) indicated a mean productivity gain of 30%, corresponding to an exploratory perceived productivity value of $6.3M to $18.9M under varying extrapolation assumptions. Although most hospital employees never used the HIPAA-compliant LLM chatbot, a small minority who utilized it regularly reported substantial productivity improvements, highlighting both the value proposition of LLMs in healthcare and potential for increased engagement. Implementation and knowledge challenges were more frequently cited than AI-feature and performance concerns as key barriers to adoption. These findings underscore that successful LLM implementation in healthcare hinges not only on advanced AI technology, but also on robust system capacity building including investments in user education and technical integration.

## Introduction

Mounting administrative burdens, burnout, and decreased face-to-face time with clinicians have spurred a growing interest in leveraging artificial intelligence within healthcare [1]. Large language models (LLMs), with their capacity to synthesize and process complex medical information, have emerged as promising tools to address some of these challenges [2]. Across the adult and pediatric healthcare industry, LLMs can be integrated to improve operational and clinical experiences [3,4]. Although consumer-facing LLM platforms such as ChatGPT were rapidly adopted to leverage generative AI, like other hospitals [5], the institution identified a need for a dedicated employee-facing AI chatbot that could handle protected health information (PHI) in a secure environment.

**Fig 1:**
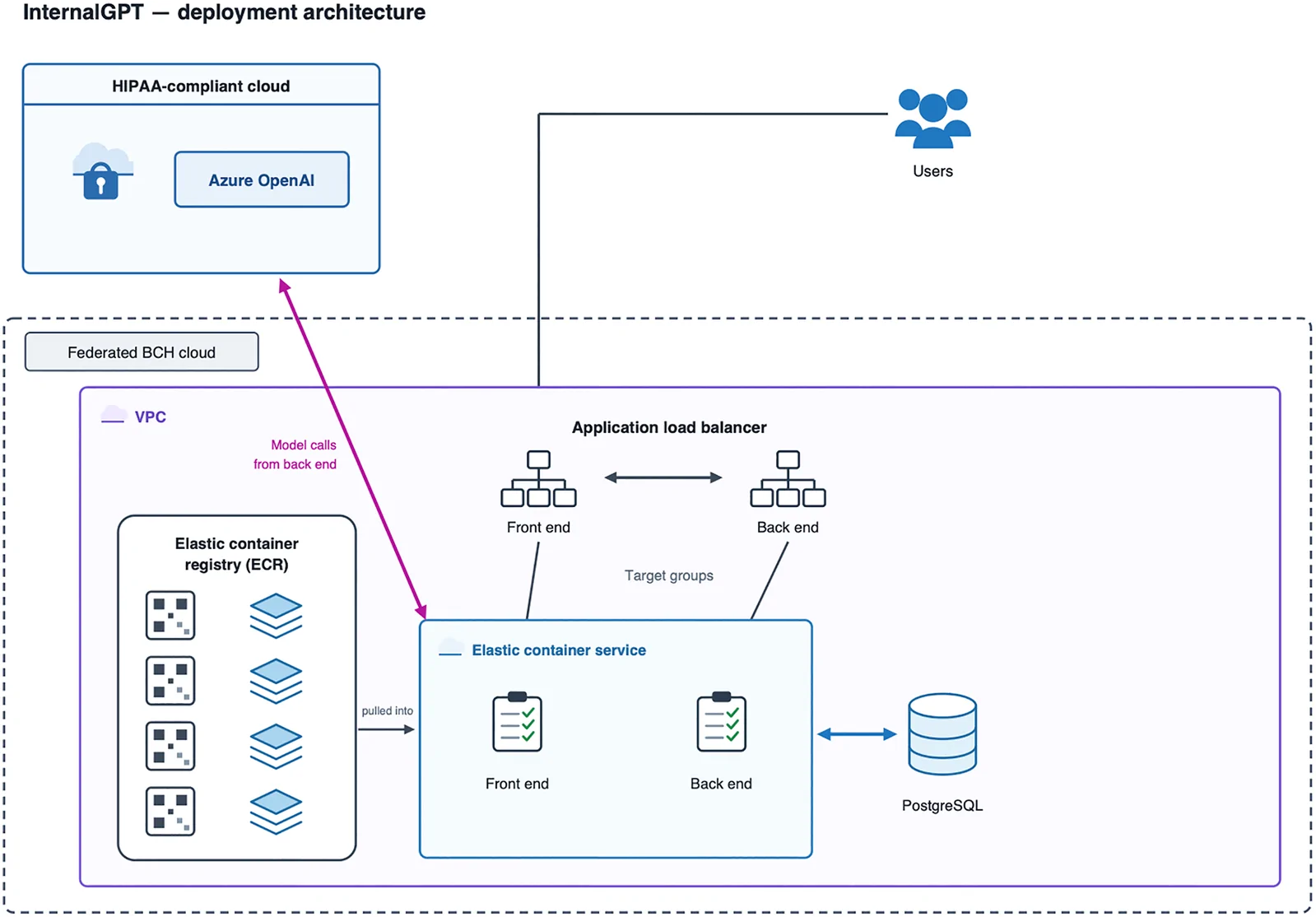
A schematic of a HIPAA-compliant LLM-chatbot for internal use at a pediatric academic medical center. A schematic of a HIPAA-compliant LLM-chatbot in use at Boston Children’s Hospital. User requests are securely directed through a traffic management system to separate web and application servers hosted on Amazon's cloud platform. The underlying AI model is provided by Azure OpenAI, and all components operate within a protected, HIPAA-compliant environment behind BCH's firewall to ensure patient data privacy and security.

In November of 2023, the academic pediatric medical center launched “InternalGPT,” a HIPAA-compliant LLM-powered chatbot that allows enterprise employees to safely explore how an LLM could enhance their work (Fig 1). Previous case studies of institutions launching similar tools have described implementation successes and challenges with deploying this type of system [5]. However, sparse data exists on hospital employee engagement with HIPAA-compliant LLM chatbots, and there remains a critical need for empirical investigations to provide concrete data on AI utilization and value within hospital systems [6].

Leveraging system utilization data and user and non-user surveys, this study examines the initial adoption and usage patterns of the InternalGPT tool across diverse professional roles within the institution. The investigation focuses on four key areas: the motivations behind user sign-up, patterns of token utilization, estimated productivity gains, and factors contributing to the discontinuation of platform use by some users. This paper shares novel qualitative and quantitative data on how clinicians, scientists, and other hospital employees utilize HIPAA-compliant LLM tools, as well as lessons learned about the adoption of AI following 14-months of InternalGPT usage at the pediatric academic medical center.

## Methods

### Platform

InternalGPT features a minimalist chat interface connected to a Microsoft Azure backend that facilitates secure and flexible access to language models via API (Fig 1). Users were generally provided access to advanced publicly released LLMs (e.g., GPT-3.5, GPT-4) with a several month lag following their availability. InternalGPT is fully integrated within the institution's HIPAA-compliant technology stack and was developed alongside a robust AI governance process. The system employs strict privacy controls (e.g., single sign-on credentials) and data security mechanisms to safeguard PHI while enabling staff to explore LLM-chatbot capabilities. Additionally, a Business Associate Agreement with Microsoft covered all data processed through Azure OpenAI Service. PHI was transmitted as-is over HTTPS-encrypted connections, and API endpoints were accessible only from the institutional VPN. Azure does not retain queries or conversation content, however the institution maintains an encrypted PostgreSQL database that stores conversation logs and access records in accordance with HIPAA requirements. To facilitate resource management, regulatory compliance, and adherence to budgetary constraints, the system automatically logs platform use and token consumption. Users were limited to 50,000–100,000 monthly tokens, a figure that shifted over time with model cost and availability changes.

### System access and motivation for adoption

The deployment of InternalGPT was announced alongside a multifaceted enterprise AI engagement and education campaign. Users were instructed to request system access via Smartsheet, a project-management focused data collection and organizational software, by providing a brief motivational statement on their intended use of the platform. Given employees at academic medical centers wear many hats, for analysis, professional roles (scientific, clinical, or operational) were assigned by GPT-4o based on job title and department (prompts in S1 Text, request form in S2 Text). These roles have substantial overlap and assignment likely includes misclassification. To identify primary objectives for InternalGPT sign-up, motivational statements were subjected to an LLM-powered qualitative thematic analysis. The analysis leveraged o3-mini to generate a list of 15 thematic categories and their definitions. That list was then submitted along with each user’s motivational statement iteratively to GPT-4o for coding. To validate LLM-assigned thematic categories, two independent human raters (one clinician [DKR], one operations professional [MLS]) hand-coded a random 100-item subset. Cohen’s kappa and percent agreement were calculated for each pairwise comparison. Agreement between the two human coders was only moderate (κ = 0.58, 63.3%), reflecting the inherently subjective nature of these thematic assignments. Agreement between each human rater and the LLM was also moderate, but as good as or better than human–human agreement (Rater 1 vs LLM: κ = 0.59, 64.3%; Rater 2 vs LLM: κ = 0.64, 68.0%).

### Data capture

Leveraging recorded system logs, an analysis was performed on utilization patterns including identifying users who requested access to the system but never logged-on or recorded a token.

### User types

**Fig 2:**
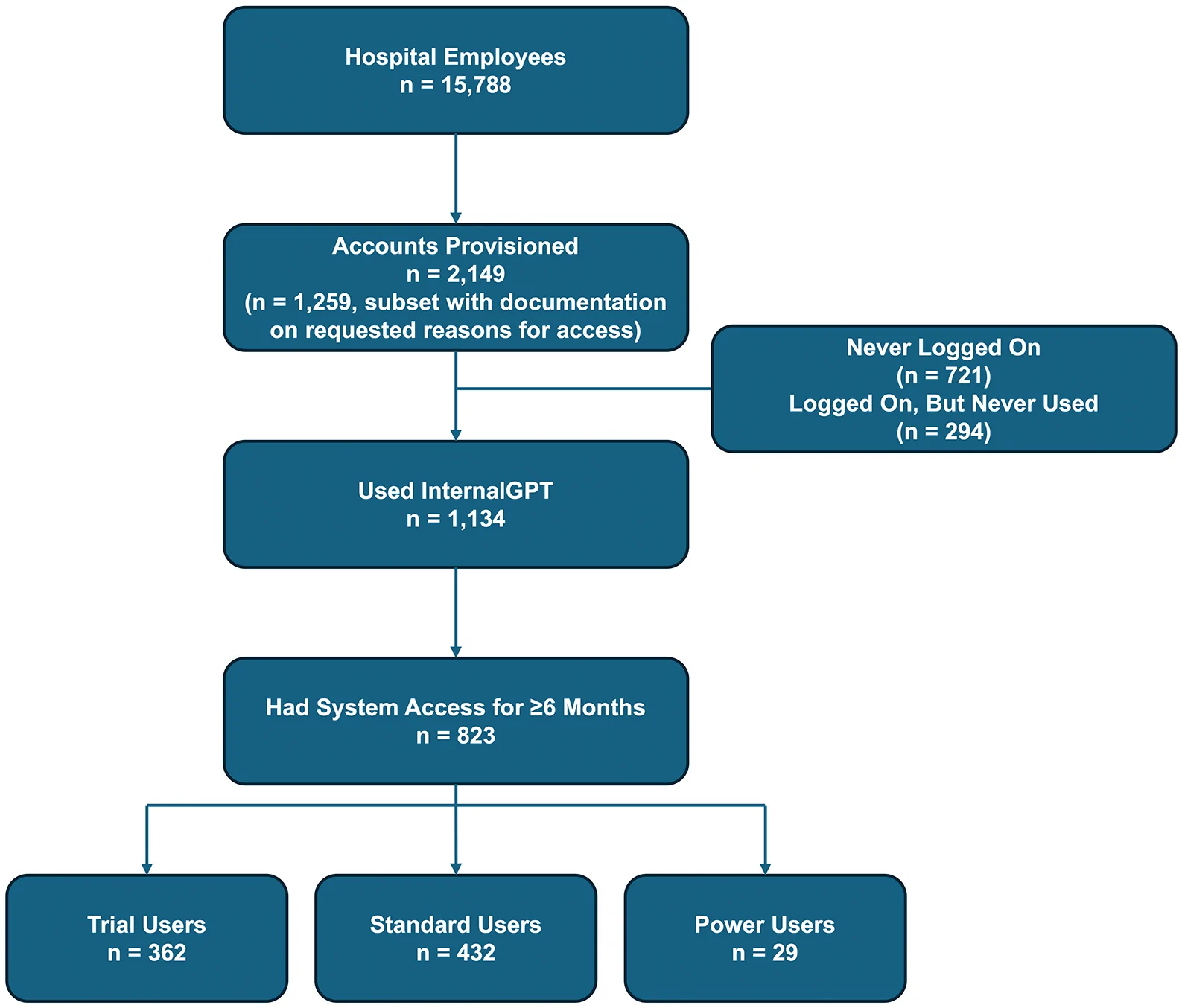
User engagement, access, and utilization flow diagram for an LLM-powered chatbot deployed at a pediatric academic medical center. Users of a HIPAA-compliant Large Language Model powered chatbot (“InternalGPT”) at a pediatric academic medical center between December 1, 2023 and January 31, 2025. All of the approximately 15,788 hospital employees were eligible to request access to InternalGPT and 2,149 did. Employees who had InternalGPT access for 6 or more months were stratified into three buckets: trial users, standard users, and power users. Trial users were defined as users who only recorded token usage in one or two months of their registered use. Power users were defined as those recording token utilization >2 standard deviations above the mean (i.e., statistical outliers). All remaining users were considered standard users.

Users who had their accounts for ≥6 months were stratified into three buckets by token utilization: trial users, standard users, and power users (Fig 2). Trial users were defined as those who recorded token usage in only one or two months since sign-up, a threshold chosen to capture users who tried the platform but did not sustain use, as three or more active months suggests at least sporadic task integration. Power users were defined as those recording token utilization >2 standard deviations above the mean (i.e., statistical outliers). Because token usage was right-skewed, a sensitivity analysis using a 95th-percentile cutoff was also performed. All remaining users were considered standard users.

### Token utilization

Temporal analysis was conducted of token use by month since sign-up. Additionally, users were sorted by token use percentile to build a Lorenz curve that describes the distribution of utilization within the population.

### System non-use and discontinuation

To understand why some users registered for InternalGPT but never actively engaged with it, and why trial users discontinued use shortly after adoption, a voluntary single-question Smartsheet survey was conducted and delivered via email. The survey queried barriers to initial engagement and factors driving discontinuation, offering structured multiple-choice options (select all that apply) and an unstructured free-text “other” field (exact survey wording in S3 Text).

### Productivity

To estimate perceived productivity gains associated with InternalGPT, it was assumed that the average hospital employee (including clinicians) cost $136,500 a year ($105k median Boston hospital pay [Glassdoor] plus 30% fringe). All standard and power users were emailed a one-question anonymous voluntary SmartSheet survey asking them to estimate their average productivity gains [-100%–100%] attributable to InternalGPT (exact wording in S4 Text). Non-users and trial users were assumed to have generated no noticeable productivity gains. To account for likely selection bias among respondents, a range of perceived value was estimated: the lower bound assumes non-respondents experienced one-sixth the gains of respondents, and the upper bound assumes non-respondents matched respondents. These estimates reflect perceived effort reduction, not measured output or cost savings.

### Additional considerations

In accordance with federal regulations and Boston Children’s Hospital guidelines, this quality improvement effort was determined by the study team not to constitute human subjects research and was not evaluated by the Institutional Review Board.

## Results

Between launch on December 1, 2023 and January 31, 2025, 2,149 users (13.6% of the approximately 15,788 hospital employees) requested and were provisioned access to InternalGPT. Data on sign-up motivation and professional role were available for 1,259 of the requestors (Table 1). The plurality (47.1%) of InternalGPT sign-ups were clinicians, followed by operations professionals (30.3%), and scientists (22.6%).

**Table 1:** Results from an AI-powered qualitative thematic analysis of the primary reasons cited for requesting access to a HIPAA-compliant LLM-powered chatbot.

	Professional Role			
**Reason for InternalGPT sign-up**	**Operations** n = 382	**Clinical** n = 593	**Scientific** n = 284	**Total** n = 1,259
AI Experimentation & Innovation	6.3%	5.1%	8.5%	6.2%
Administrative Workflow Automation	55.8%	21.1%	19.7%	31.3%
Clinical Decision Support	0.5%	6.2%	0.4%	3.2%
Clinical Documentation Automation	3.1%	17.4%	5.3%	10.3%
Coding and Data Assistance	7.1%	3.7%	21.5%	8.7%
Financial Billing Analytics	2.1%	0.0%	0.0%	0.6%
Healthcare Data Integration	0.5%	1.9%	2.8%	1.7%
Language Translation Simplification	2.4%	3.0%	2.1%	2.6%
Medical Education Materials	4.7%	10.3%	2.8%	6.9%
Patient Letter Generation	0.8%	16.4%	2.1%	8.4%
Project Management Summaries	1.8%	0.3%	0.7%	0.9%
Quality Improvement Analytics	5.5%	1.2%	0.7%	2.4%
Regulatory Policy Documentation	4.2%	2.0%	3.2%	2.9%
Research Literature Summarization	5.2%	9.3%	29.9%	12.7%
Patient Care Treatment Planning	0.0%	2.2%	0.4%	1.1%

The most common motivation for requesting access was administrative automation, which was heavily cited across all roles (31.3% of total sign-ups) including 55.8% of operations professionals. Medical purposes including clinical documentation automation (17.4%), patient letter generation (16.4%), medical education materials (10.3%), clinical decision support (6.2%), and patient care treatment planning (2.2%) made up the majority (52.5% total) of reasons cited by clinicians. Scientists heavily cited research tasks including research literature summarization (29.9%), coding & data assistance (21.5%), and AI experimentation & innovation (8.5%).

Many employees who requested LLM system access did not utilize the tool including 721 employees (33.6% of accounts provisioned) who never logged into the system and 294 employees (13.7%) who logged in, but never recorded a token use (Fig 2). The slight majority (52.8%, n = 1,134) of requesters did use the system at least once, had account access for a mean of 8.8 months, and recorded a monthly mean of 6,363.3 tokens (SD: 16,894.1). During the study period, 75 individual monthly cap-hit events occurred across approximately 59 unique users; however, token limits were increased on a case-by-case basis for users who consistently reached their caps.

Token utilization patterns were analyzed for the 823 users who used InternalGPT and had account access for ≥6 months. Trial users who recorded tokens in only one or two months since sign-up (n = 362) made up 44.0% of users (Fig 2). The remaining users were split into power users (n = 29, 3.5%) and standard users (n = 432, 52.5%). Trial users were slightly less likely to be clinicians (44.8% versus 47.1% of sign-ups) and more likely to be scientists (30.3% versus 22.6% of sign-ups). Operations professionals were disproportionately likely to be power users (46.2% versus 30.3% of sign-ups).

**Fig 3:**
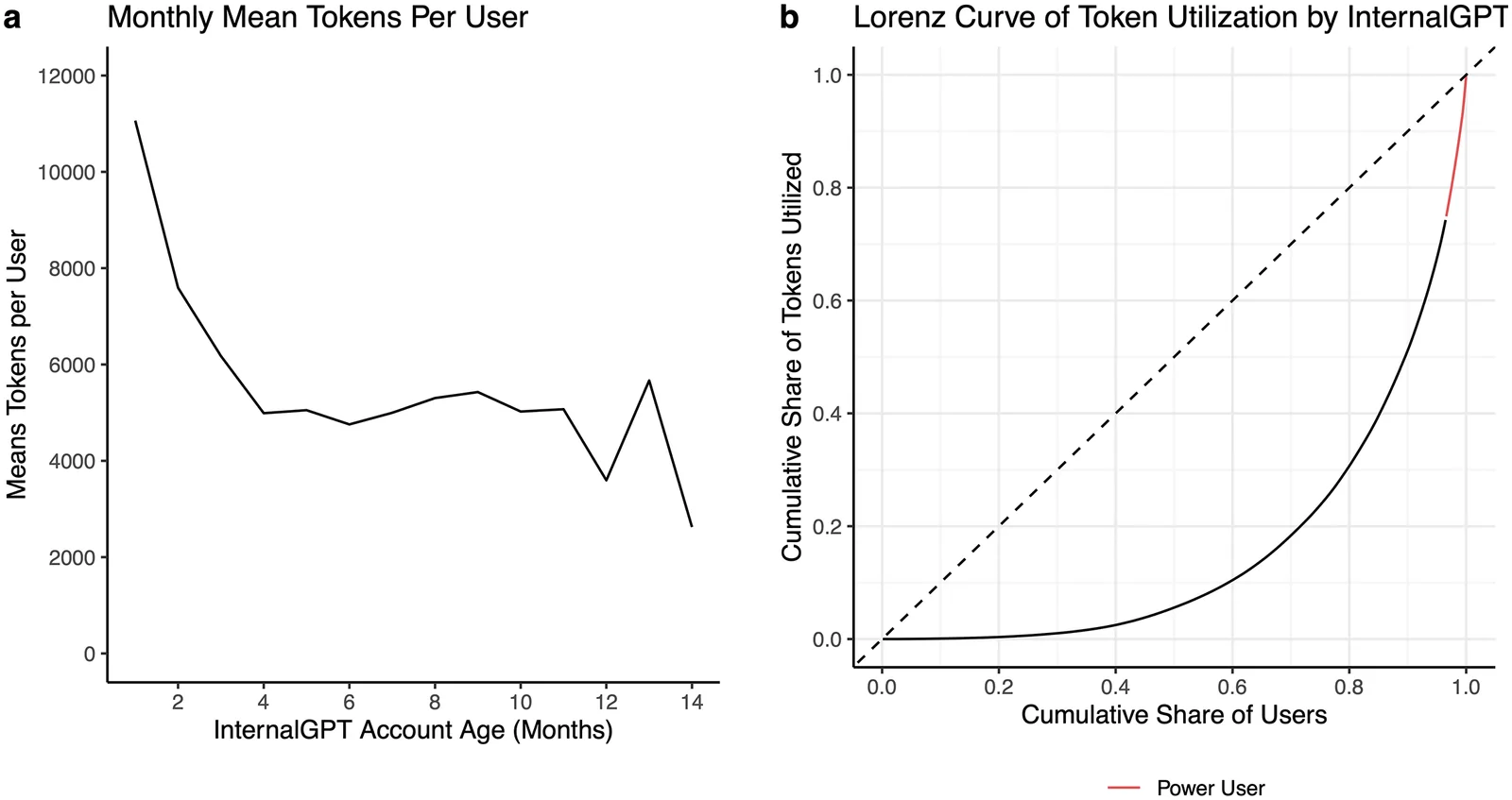
User level token utilization of an LLM-powered chatbot deployed at a pediatric academic medical center between December 1, 2023 and January 31, 2025. Token utilization patterns of n = 1,134 users of HIPAA-compliant LLM-powered chatbot, including average monthly tokens consumed per user by months since first use (a) and Lorenz curve of cumulative token consumption (b). The bottom 50% of users account for only 5.6% of tokens used, while the top 20% consume 69.4%, including power users (the top 3.5%) who were responsible for 25.8%.

Among all users, token usage initially dropped by month since first-use but later stabilized (Fig 3a) and was skewed with a small percentage of users making up the majority of tokens consumed (Fig 3b). The bottom 50% of users utilized just 5.6% of tokens, while the top 20% utilized 69.4% including the power users (i.e., statistical outliers) who utilized 25.8%. A sensitivity analysis utilizing a 95th-percentile cutoff produced similar results, identifying 24 power users (versus 29 under the base definition) who consumed approximately 22% of tokens, justifying the broad conclusion that a small number of users consumed the majority of the tokens.

Trial users and employees who signed up for InternalGPT but did not use it were surveyed on reasons for their lack-of or reduction in use. Among the 142 responses (10.3% response rate), the most common endorsed reasons were lacking time to explore/evaluate (51.4%) and having trouble figuring out how to use (21.8%), integrate (15.5%), or access (9.2%) InternalGPT (Table 2).

**Table 2:** Results from a survey (n = 142) on why healthcare system employees did not use or later reduced their usage of a HIPAA-compliant LLM-powered chatbot.

Which of the following reasons explains why you did not or stopped using InternalGPT?	Percentage Endorsed
I have not had time to explore and evaluate InternalGPT yet	51.4%
I could not figure out how to use InternalGPT	21.8%
I struggle to integrate InternalGPT into my workflow	15.5%
I find InternalGPT too difficult/tedious to access	9.2%
I have not been allocated enough tokens for my needs	8.5%
I require an AI tool with more/different features than InternalGPT currently offers	6.3%
I only signed up for InternalGPT to see what the hype was about	5.6%
I require an AI tool with higher response quality than InternalGPT	5.6%
Technical issues have impacted my ability to use InternalGPT	5.6%
Ethical concerns about InternalGPT have impacted my use of the tool	4.9%
InternalGPT did not solve my problem and/or I did not find the output from InternalGPT useful	4.2%
Compliance/regulatory concerns about InternalGPT have impacted my use of the tool	3.5%
I require an AI tool that is powered by a different LLM than InternalGPT is	3.5%
I require an AI tool with faster response speed than InternalGPT	3.5%
What is InternalGPT?	2.1%
Large Language Models like those powering InternalGPT do not solve my problems	1.4%
Other*	38.7%

*Common themes among the individuals who selected “other” included not requiring a HIPAA-compliant model, or having access to an alternative AI platform. Multiple users also expressed token anxiety and used words like “stressed” and “worried” to describe feeling like they may hit their quota.

Common themes among the individuals who selected “other” included not requiring a HIPAA-compliant model or having access to an alternative AI platform. Multiple users also expressed token anxiety and used words like “stressed” and “worried” to describe feeling like they may exhaust their quota.

Standard and power user survey respondents (n = 92, 20.0% response rate) estimated they gained a mean 30% productivity [SD:28.1%] with the use of InternalGPT, a figure consistent with prior estimates [7]. Standard and power user survey non-respondents were assumed to gain between 1/6^th^ (5%) and the full (30%) productivity estimates of survey respondents. Notably, these estimates do not factor-in trial users and non-users who likely gained 0% productivity. Assuming an average employee cost of $136,500 per year, estimated perceived productivity value gained ranged from $13,636 to $40,950 per standard/power user, yielding $6.3M to $18.9M across all 461 standard/power users depending on assumptions about non-respondent gains. These figures reflect self-reported perceived effort reduction, not demonstrated cost savings or measured output. Across the entire hospital system, these gains correspond to $2,925–$8,785 per account provisioned or $398–$1,196 per employee offered access.

## Lessons learned

1. The majority of individuals offered access to InternalGPT realized no value; they either never accessed the tool or tried it and ceased use. However, for the small portion of the hospital system who gained access to the system and used it consistently, the self-reported productivity gains were tremendous and were large enough to justify broad enterprise access to a HIPAA-compliant LLM chatbot.
2. The asymmetry of InternalGPT use was also reflected within the users of the tool. In the Lorenz curve of token utilization, the data revealed a pattern that approached the classic 80/20 Pareto principle; the top 20% of users consumed approximately 70% of tokens including a group of power users who represented <4% of the user base but >25% of tokens.
3. In general, hospital professionals were primarily motivated to use InternalGPT to improve core aspects of their job: clinicians wanted to use LLMs to augment clinical tasks, scientists wanted to use LLMs to aid in research, and operations professionals wanted to use LLMs to improve administrative workflows. While there was broad interest in automating administrative tasks across all professional roles, these findings stand in contrast to the occasional assumption that individuals primarily hope to use AI to automate peripheral or convenience tasks, rather than assist with essential job functions. These motivations suggest potential for improved clinical care delivery.
4. Despite high initial interest in InternalGPT, large gaps remain between curiosity and sustained engagement. Substantially more users cited access and integration challenges than concerns about model performance or functionality. While a minority of power users may benefit from investment in advanced tools and cutting-edge models for LLM chatbots, more basic technical infrastructure challenges emerged as a primary barrier for broader adoption.
5. Successful deployment of AI tools requires more than the technology itself. To effectively address barriers such as difficulty learning how to use InternalGPT, substantial investments in employee capacity building are required. Although InternalGPT aimed to enhance productivity and save time, many employees indicated they lacked sufficient time to effectively learn to leverage the tool, reflecting a meaningful learning curve. Addressing this paradox will be crucial for realizing the full potential of LLMs. Efforts to integrate user-friendly guidance directly into InternalGPT (e.g., prompt libraries, guide modes, and assistants) are being piloted to mitigate these challenges.
6. While cost containment features may be important to prevent token hogs and nonstandard applications, the monthly average use of the tool fell far below utilization limits and those who used the tool regularly self-report large productivity gains - suggesting value commensurate with use. Additionally, token quota anxiety may prevent users from fully taking advantage of LLMs.

## Limitations

InternalGPT offers reduced functionality compared to commercial alternatives (e.g., ChatGPT), lacking features such as internet search, which, alongside token caps, may have constrained its utility and adoption. Moreover, the analysis did not capture consumer LLM data, which many employees may use concurrently or alternatively. InternalGPT’s launch coincided with the enterprise rollout of a new EMR and the deployment of other AI solutions (e.g., AI-powered scribes), potentially introducing technology adoption fatigue. Additionally, the analysis may only offer a partial view of the organization's HIPAA-compliant LLM usage patterns, as small-scale research LLM instances and API-based tools were being piloted throughout the hospital concurrent with InternalGPT and may include many power users [8]. The surveys also likely suffer from non-response bias with the users who are most burned out and/or find the least value in InternalGPT also being the least likely to reply. The voluntary nature of InternalGPT adoption limits generalizability of utilization patterns. Token quotas may have capped observed demand among the heaviest users, meaning the true utilization distribution could be even more skewed. The productivity survey’s 20% response rate likely overrepresents satisfied users; a sensitivity analysis allowing for differences between respondents and non-respondents attempts to account for this but remains exploratory. Professional role assignment by LLM is approximate given that many employees span multiple categories and no institutional gold-standard classification exists. Imperfect data collection timing and efforts reflect the real-world implementation described in this case study; however, future studies would benefit from a research design from the outset, such as randomizing access and measuring benefit via intent-to-treat. Lastly, perceived productivity gains may not be empirically rooted or translate to institutional gains; however, perceived workload is an important predictor of burnout [9].

## Conclusion

Despite modest uptake, deployment of a HIPAA‑compliant LLM chatbot at an academic pediatric medical center delivered self-reported productivity and workflow benefits which could be improved upon with strong AI capacity-building efforts.

## Supporting information

S1 Text LLM prompts used for role assignment and thematic categorization. (DOCX)

S2 Text InternalGPT access request form. (DOCX)

S3 Text Non-use and discontinuation survey. (DOCX)

S4 Text InternalGPT productivity survey. (DOCX)

## References

[1] Topaz M, Peltonen LM, Zhang Z. Beyond human ears: navigating the uncharted risks of AI scribes in clinical practice. NPJ Digit Med. 2025;8(1):569. doi: 10.1038/s41746-025-01895-6 40993221 PMC12460601

[2] Peng C, Yang X, Chen A, Smith KE, PourNejatian N, Costa AB, et al. A study of generative large language model for medical research and healthcare. NPJ Digit Med. 2023;6(1):210. doi: 10.1038/s41746-023-00958-w 37973919 PMC10654385

[3] Christakis DA, Neely J, Spalding W, Opel DJ. How artificial intelligence can promote inclusive health. JAMA Pediatr. 2025. doi: 10.1001/jamapediatrics.2025.0096 40094693

[4] Goodell AJ, Chu SN, Rouholiman D, Chu LF. Large language model agents can use tools to perform clinical calculations. NPJ Digit Med. 2025;8(1):163. doi: 10.1038/s41746-025-01475-8 40097720 PMC11914283

[5] Umeton R, Kwok A, Maurya R, Leco D, Lenane N, Willcox J, et al. GPT-4 in a cancer center — institute-wide deployment challenges and lessons learned. NEJM AI. 2024;1(4). doi: 10.1056/aics2300191

[6] Kohane IS. Compared with what? Measuring AI against the health care we have. N Engl J Med. 2024;391(17):1564–6. doi: 10.1056/NEJMp2404691 39465890

[7] Noy S, Zhang W. Experimental evidence on the productivity effects of generative artificial intelligence. Science. 2023;381(6654):187–92. doi: 10.1126/science.adh2586 37440646

[8] Akhondi-Asl A, Yang Y, Luchette M, Burns JP, Mehta NM, Geva A. Comparing the quality of domain-specific versus general language models for artificial intelligence-generated differential diagnoses in PICU patients. Pediatr Crit Care Med. 2024;25(6):e273–82. doi: 10.1097/PCC.0000000000003468 38329382

[9] Meredith LS, Bouskill K, Chang J, Larkin J, Motala A, Hempel S. Predictors of burnout among US healthcare providers: a systematic review. BMJ Open. 2022;12(8):e054243. doi: 10.1136/bmjopen-2021-054243 36008065 PMC9422884

