# Supplementary material for: Utilization of a HIPAA-compliant large language model chatbot in an academic pediatric medical center
Source: PLOS Digit Health. 2026 Sep 17;5(9):e0001684. doi: 10.1371/journal.pdig.0001684 (PMC13585266; doi:10.1371/journal.pdig.0001684)
Supplement: Supplementary file 1 [file pdig.0001684.s001.docx]

*Utilization of a HIPAA-Compliant Large Language Model Chatbot in an Academic Pediatric Medical Center*

## S1 Text. LLM prompts used for role assignment and thematic categorization.

Three LLM prompts were used in this study for qualitative coding: (a) a role assignment prompt used to classify employees into professional roles, (b) a categories prompt used to generate the thematic taxonomy, and (c) a categorization prompt used to assign each motivational statement to a thematic category.

**Prompt A. Role Assignment Prompt (GPT-4o)**

*You are an expert at classifying staff roles. For each staff member, you will be given a Department and Position.*

*You will need to choose one of the following categories exactly: "Scientific", "Clinical", "Operations".*

*If the position or department is nan, return "N/A"*

*Here are some examples of Clinical Staff:*
*- Doctor*
*- Nurse*
*- Allied Health Professional*
*- Pharmacist*
*- Resident*
*- Fellow*
*- Other trainee*
*- Geneticist*
*- Radiologist*
*- Other clinical staff*

*Here are some examples of Scientific Staff:*
*- Scientist*
*- Researcher*
*- Lab Technician*
*- Bioinformatician*
*- Bioengineer*
*- Biologist*
*- Other scientific staff*

*Here are some examples of Operations Staff:*
*- Administrator*
*- Operations Manager*
*- Receptionist*
*- Cleaner*
*- Security Guard*
*- Maintenance Staff*
*- IT Staff*
*- Finance Staff*
*- HR Staff*
*- Management Staff*

**Prompt B. Categories Prompt (o3-mini)**

*You are a senior taxonomy architect specializing in generative-AI use cases for hospitals.*

*Task*
*Create a complete taxonomy of 15 high-level, mutually exclusive categories to classify the free-text "intended-use" statements that follow.*

*Mandatory Rules*
*Distinct -- Design boundaries so each use case fits into one and only one category. The wording of every category must make its unique scope obvious.*
*Complete -- Together the categories must cover the entire set. If, after careful review, you cannot guarantee coverage with 14 precise categories, you may allocate exactly one slot to an "Other" category.*

*Title -- 3-5 plain-English words, Upper Camel Case, noun-based (no verbs).*
*Description -- 2-4 concise sentences in plain English. State the typical output, primary audience, and purpose. End with a sentence that makes it clear why this category is not similar to any other (e.g., key audience difference, legal status, or intent).*

*Disambiguation Guide -- Before finalizing, inspect your draft list for overlaps. Refine wording so every pair differs by at least one decisive attribute. Do not mention category names here; just ensure your language prevents ambiguity.*

*Output Format -- Return only valid JSON structured exactly as follows:*
*{*
 *"categories": [*
 *{ "name": "<3-5 word title>", "description": "<2-4 sentence description>" },*
 *... (total 15 objects) ...*
 *]*
*}*

*Quality Check -- Re-read your list. If any overlap remains or the count is not 15, fix it before responding.*

*Context*
*These categories will power automated tagging of thousands of staff requests for a healthcare-focused chat interface.*

*Submitted User Use Cases:*
*[use cases provided here]*

**Prompt C. Categorization Prompt (GPT-4o)**

```
You are an expert in categorizing healthcare AI use cases. Analyze each use case and assign the most appropriate category from the provided codebook. Be precise in matching the exact category names provided. Respond in JSON format with the structure:

{
  "category": "Category Name",
  "explanation": "Brief explanation of why this category was chosen"
}

Based on the following codebook, categorize this use case:

Use case: {use_case}

{codebook_text}

Provide:
1. The exact name of the most appropriate category from the codebook
2. A brief explanation of why you chose this category
```
