# Supplementary material for: Utilization of a HIPAA-compliant large language model chatbot in an academic pediatric medical center
Source: PLOS Digit Health. 2026 Sep 17;5(9):e0001684. doi: 10.1371/journal.pdig.0001684 (PMC13585266; doi:10.1371/journal.pdig.0001684)
Supplement: Supplementary file 2 [file pdig.0001684.s002.docx]

*Utilization of a HIPAA-Compliant Large Language Model Chatbot in an Academic Pediatric Medical Center*

## S2 Text. InternalGPT access request form.

Employees requesting access to InternalGPT were directed to complete the following SmartSheet form.

***BCH ChatGPT Access Request Form***

**Name** *– Required*

**Email** *– Required. The email provided should be one of the BCH domain (@chtrust.org, @childrens.harvard.edu, or @cardio.chboston.org)*

**Department** *– Required. Free response.*

**Intended Use** *– Required. “Describe how you plan to use InternalGPT (i.e. example use case(s)).” Free response.*

**Primary Subject Matter** *– Required. “Will you primarily be using InternalGPT for administrative tasks, research purposes, or inputting clinical data?”*

• *Admin*

• *Medical/Clinical*

• *Research*

**Anticipated Frequency of Use** *– Required.*

• *Low Volume Experimentation (i.e. up to a couple of times a week)*

• *Moderate Volume (i.e. up to a couple of times daily)*

• *High Volume (i.e. multiple times daily)*

**Terms of Use Agreement** *– Required. “I agree to review the BCH InternalGPT Terms of Use when granted access to the application.”*
