# Supplementary material for: Utilization of a HIPAA-compliant large language model chatbot in an academic pediatric medical center
Source: PLOS Digit Health. 2026 Sep 17;5(9):e0001684. doi: 10.1371/journal.pdig.0001684 (PMC13585266; doi:10.1371/journal.pdig.0001684)
Supplement: Supplementary file 3 [file pdig.0001684.s003.docx]

*Utilization of a HIPAA-Compliant Large Language Model Chatbot in an Academic Pediatric Medical Center*

## S3 Text. Non-use and discontinuation survey.

Trial users and employees who signed up for InternalGPT but did not use it were surveyed on reasons for their lack of or reduction in use. The following voluntary single-question SmartSheet survey was delivered via email.

**Survey text:**

*To improve the quality of our AI offerings, we are collecting data on why users signed up for BCH’s HIPAA compliant instance of ChatGPT (InternalGPT) and did not use the service or later reduced their usage.*

***Which of the following reasons explains why you did not or stopped using the InternalGPT?***

*Please select all that apply*

• *What is InternalGPT?*

• *I could not figure out how to use InternalGPT*

• *I only signed up for InternalGPT to see what the hype was about*

• *I have not had time to explore and evaluate InternalGPT yet*

• *InternalGPT did not solve my problem and/or I did not find the output from InternalGPT useful*

• *Large Language Models like those powering InternalGPT do not solve my problems*

• *I find InternalGPT too difficult/tedious to access*

• *I require an AI tool with higher response quality than InternalGPT*

• *I require an AI tool that is powered by a different LLM than InternalGPT is*

• *I require an AI tool with faster response speed than InternalGPT*

• *I require an AI tool with more/different features than InternalGPT currently offers*

• *I struggle to integrate InternalGPT into my workflow*

• *I have not been allocated enough tokens for my needs*

• *Technical issues have impacted my ability to use InternalGPT*

• *Compliance/regulatory concerns about InternalGPT have impacted my use of the tool*

• *Ethical concerns about InternalGPT have impacted my use of the tool*

• *Other*
