# Supplementary material for: Utilization of a HIPAA-compliant large language model chatbot in an academic pediatric medical center
Source: PLOS Digit Health. 2026 Sep 17;5(9):e0001684. doi: 10.1371/journal.pdig.0001684 (PMC13585266; doi:10.1371/journal.pdig.0001684)
Supplement: Supplementary file 4 [file pdig.0001684.s004.docx]

*Utilization of a HIPAA-Compliant Large Language Model Chatbot in an Academic Pediatric Medical Center*

## S4 Text. InternalGPT Productivity Survey

Standard and power users were emailed the following one-question anonymous voluntary SmartSheet survey asking them to estimate their average productivity gains [–100% to 100%] attributable to InternalGPT.

**Survey text:**

*To improve the quality of our AI offerings, we are collecting data on BCH’s HIPAA compliant instance of ChatGPT (InternalGPT).*

***InternalGPT Impact****
*By filling in the blank in the sentence below, how would you describe the impact of InternalGPT on your overall productivity? Please provide an anonymous estimate of any improvements in your daily or monthly work output, expressed as a percentage.*

*On average, I am ___% more productive with InternalGPT. [-100%,100%]*
